# Regulation of bone phosphorus retention and bone development possibly by related hormones and local bone-derived regulators in broiler chicks

**DOI:** 10.1186/s40104-021-00610-1

**Published:** 2021-08-12

**Authors:** Sumei Cao, Tingting Li, Yuxin Shao, Liyang Zhang, Lin Lu, Rijun Zhang, Shuisheng Hou, Xugang Luo, Xiudong Liao

**Affiliations:** 1 Mineral Nutrition Research Division,State Key Laboratory of Animal Nutrition, Institute of Animal Science, Chinese Academy of Agricultural Sciences, Beijing, 100193 People’s Republic of China; 2 Poultry Mineral Nutrition Laboratory, College of Animal Science and Technology, Yangzhou University, Yangzhou, 225000 People’s Republic of China; 3 Laboratory of Feed Biotechnology, State Key Laboratory of Animal Nutrition, College of Animal Science and Technology, China Agricultural University, Beijing, 100193 People’s Republic of China

**Keywords:** Bone, Broiler chicken, Hormone, Local bone-derived regulator, Phosphorus

## Abstract

**Background:**

Phosphorus is essential for bone mineralization in broilers, however, the underlying mechanisms remain unclear. We aimed to investigate whether bone phosphorus retention and bone development might be regulated by related hormones and local bone-derived regulators in broilers.

**Methods:**

Broilers were fed diets containing different levels of non-phytate phosphorus (NPP) 0.15%, 0.25%, 0.35%, 0.45% and 0.55% or 0.15%, 0.22%, 0.29%, 0.36% and 0.43% from 1 to 21 or 22 to 42 days of age. Serum and tibia samples were collected for determinations of bone phosphorus retention and bone development parameters, related hormones and local bone-derived regulators of broiler chickens on d 14, 28 and 42, respectively.

**Results:**

Tibia ash phosphorus, total phosphorus accumulation in tibia ash (TP_TA_), bone mineral concentration (BMC), bone mineral density (BMD), bone breaking strength (BBS), and ash on d 14, 28 or 42, serum 1,25-dihydroxyvitamin D_3_ (1,25(OH)_2_D_3_) on d 28 and 42, mRNA expressions of tibia fibroblast growth factor 23 (*FGF23*) and dentin matrix protein 1 (*DMP1*) on d 14 and 28 increased linearly or quadratically (*P* < 0.05), while serum parathyroid hormone (PTH) on d 28, tibia alkaline phosphatase (ALP) on d 14, 28 and 42, bone gal protein (BGP) on d 14, and mRNA expression of tibia phosphate-regulating gene with homologies to endopeptidases on the X chromosome (*PHEX*) on d 14 and 28 decreased linearly or quadratically (*P* < 0.04) as dietary NPP level increased. TP_TA,_ BMC, BMD, and ash on d 28 and 42, BBS on d 28, and ash phosphorus on d 42 were positively correlated (*r* = 0.389 to 0.486, *P <* 0.03) with serum 1,25(OH)_2_D_3_. All of the above parameters were positively correlated (*r* = 0.380 to 0.689, *P <* 0.05) with tibia *DMP1* mRNA expression on d 14, 28 and 42, but negatively correlated (*r* = − 0.609 to − 0.538, *P <* 0.02) with serum PTH on d 28, tibia ALP on d 14, 28 and 42, and BGP on d 14. TP_TA_, BMC and ash on d 14 and BMD on d 28 were negatively correlated (*r* = − 0.397 to − 0.362, *P <* 0.03) with tibia *PHEX* mRNA expression, and BMD on d 28 was positively correlated (*r* = 0.384, *P =* 0.04) with tibia *FGF23* mRNA expression.

**Conclusions:**

These results suggested that bone phosphorus retention and bone development parameters had moderate to strong correlations with serum PTH and 1,25(OH)_2_D_3_ and tibia DMP1, PHEX, FGF23, ALP and BGP in broilers during the whole growth period, and thus they might be partly regulated by these related hormones and local bone-derived regulators.

**Supplementary Information:**

The online version contains supplementary material available at 10.1186/s40104-021-00610-1.

## Background

Phosphorus, in the form of inorganic phosphorus, is an essential mineral element and plays an important role in multiple biological processes, especially for bone mineralization in humans and animals [1, 2]. Many clinical researches have demonstrated that phosphorus is one of the major factors in the maintenance of bone development and health, and the deficiency of phosphorus could result in bone pathology and clinical illness [3, 4]. Besides, broiler chickens are fast-growing birds and susceptible to dietary phosphorus deficiency, and early fast growth rate is generally accompanied by a high incidence of skeletal disorders, such as hypophosphatemic rickets and tibial dyschondroplasia, partly due to an insufficient formation of hydroxyapatite in the bone of broiler chickens [5]. Thus, broiler chickens might be an ideal animal model for studying the bone development and health, and it is necessary to study bone phosphorus retention and bone development for better improving phosphorus utilization and homeostasis in the bone of broiler chicken model. The phosphorus homeostasis is achieved by complex interactions between bone and multiple organs via many hormones including but not limited to parathyroid hormone (PTH), 1, 25-dihydroxyvitamin D_3_ (1,25(OH)_2_D_3_) and fibroblast growth factor 23 (FGF23), and most of these insights are derived from human genetic disorders and genetically engineered mice [6, 7]. Furthermore, many recent studies have shown that local bone-derived factors, such as FGF23, phosphate-regulating gene with homologies to endopeptidases on the X chromosome (PHEX), matrix extracellular phosphoglycoprotein (MEPE) and dentin matrix protein 1 (DMP1), constitute a novel hormone/enzyme/extracellular matrix protein axis that regulates phosphorus homeostasis and bone mineralization [8–10]. Additionally, bone is a living tissue that is constantly changing and adapting during growth by osteoblasts and osteocytes, and osteoblasts is usually considered as a major factor regulating the skeleton growth, remodeling, and mineralization [11, 12]. It is crucial to maintain adequate inorganic phosphorus levels in the process of matrix mineralization by osteoblasts and osteocytes, and alkaline phosphatase (ALP) and bone gal protein (BGP) are also involved in the process of bone formation and matrix mineralization [13, 14]. It is thus a complex interaction process between the related hormones and local bone-derived factors for the regulation of bone phosphorus retention and bone development in the bone and needs to be further studied with animal models. However, only a few studies have been conducted to evaluate the regulation of bone phosphorus retention and bone development by FGF23 in chickens [15–17]. A recent study from our laboratory showed that the bone phosphorus retention was involved in the bone development of broilers fed the diets containing the fixed levels of non-phytate phosphorus (NPP) at different ages during d 1 to 42 possibly via the regulation of the bone ALP and BGP [12]. To our knowledge, no study has been undertaken to investigate how PHEX, MEPE, DMP1, ALP and BGP regulate bone phosphorus retention and bone development of broiler chickens fed diets containing different NPP levels from 1 to 42 days of age. Therefore, possible mechanisms of bone phosphorus retention and bone development of broilers during the whole growth period remain unclear. It was hypothesized that bone phosphorus retention and bone development of broiler chickens during the whole growth period might be regulated not only by the related hormones, but also by the local bone-derived factors in broiler chickens. The objective of the present study was to investigate the effect of dietary different levels of NPP on the growth performance, bone phosphorus retention and bone development parameters, related hormones and local bone-derived regulators in the fast-growing broiler chicken model to test the above hypothesis.

## Materials and methods

All experimental procedures were approved by the Animal Management Committee (in charge of animal welfare) of the Institute of Animal Science, Chinese Academy of Agricultural Sciences (IAS-CAAS, Beijing, China) and performed in accordance with the guidelines. Ethical approval on animal survival was given by the animal ethics committee of IAS-CAAS. We have followed the ARRIVE guidelines for reporting animal research [18].

### Animals

A total of 800 one-d-old Arbor Acres male broiler chicks (Huadu Broiler Breeding Corp., Luanping, China) with similar body weight were randomly allotted to 1 of 5 treatments with 8 replicate cages of 20 chicks per replicate cage. The birds were housed in electrically heated, thermostatically controlled cages (190 cm × 100 cm × 75 cm, length × width × height) equipped with the plastic net floor and fiberglass feeders (180 cm, length) and water supply in an environmentally controlled room with continuous 18 h of light and 6 h of dark. Environmental temperature was maintained at 35 °C for the first 3 d, after which the temperature was gradually reduced by 3 °C per week until it reached 24 °C and then was maintained at this temperature until the end of the experiment. The light intensity of 20 lx was used during the entire experimental period. Feed and tap water were provided ad libitum. Mortality was recorded daily, and chick weight and feed intake per cage were measured at 21 and 42 days of age to calculate average daily gain (ADG), average daily feed intake (ADFI), and feed conversion ratio (FCR).

### Experimental design and treatments

A completely randomized design was used in this experiment. There were a total of 5 treatments of dietary NPP levels, including 0.15%, 0.25%, 0.35%, 0.45% (control group) and 0.55% or 0.15%, 0.22%, 0.29%, 0.36% (control group) and 0.43% for broilers from 1 to 21 days of age or 22 to 42 days of age according to the National Research Council (NRC, 1994) requirements of broilers for dietary NPP levels (d 1–21: 0.45%, and d 22–42: 0.35%) [19], respectively.

### Experimental diets

The basal corn-soybean meal diets were formulated to meet or exceed the nutrient requirements of starter broilers (d 1 to 21) and grower broilers (d 22 to 42) as recommended by the Feeding standard of chicken (China, NY/T 33–2004, 2004) [20], except for calcium (Ca) and phosphorus. Dietary treatments included the basal diet (containing 0.15% NPP) supplemented with 0, 0.10%, 0.20%, 0.30% or 0.40% inorganic phosphorus in the form of feed grade CaHPO_4_^**.**^H_2_O from 1 to 21 days of age, and supplemented with 0, 0.07%, 0.14%, 0.21% or 0.28% inorganic phosphorus from 22 to 42 days of age, respectively. A single large batch of mash basal diet, void of CaHPO_4_^**.**^H_2_O and limestone, was mixed at first, and then divided into 5 sublots according to the above experimental treatments. Each supplemental CaHPO_4_^**.**^H_2_O and limestone were mixed with fine sand to the same weight [21, 22] according to the above experimental design and then mixed with each aliquot of the basal diet. Fine sand was washed several times before use, and contained no detectable phosphorus and Ca. All diets were fed in mash form. Dietary composition and nutrient levels are all expressed on an as-fed basis and are presented in Supplementary Table S1.

### Sample collections and preparations

The feed ingredients and diets from the 5 treatments were collected for analyses of crude protein (CP), Ca and phosphorus before the initiation of the experiment to confirm CP, Ca, and phosphorus concentrations in diets. At 14, 28, and 42 days of age, 4, 3 or 2 birds respectively from each replicate cage were selected according to average body weight of each cage after a 12-h fast. Blood samples were taken from the selected birds via the wing vein, immediately placed on ice, and then centrifuged at 3,000×*g* for 10 min at 4 °C to isolate serum, and then serum aliquots were stored at − 20 °C until analyses. The selected birds were then killed by cervical dislocation, the right tibia was peeled and frozen at − 20 °C and the left tibia was peeled and frozen in liquid nitrogen and then stored at − 80 °C until analyses. To reduce individual biological variation, the samples from selected birds in each replicate cage were pooled into 1 sample in equal ratios before analyses. Therefore, the number of replication was normally 8 (*n* = 8) for each parameter measurement of each treatment.

### Determination of ca, phosphorus and CP concentrations

The Ca concentrations in feed ingredient, diet and tibia ash samples were measured using an inductively coupled plasma emission spectroscope (model IRIS Intrepid II, Thermal Jarrell Ash, Waltham, MA) after wet digestions with HNO_3_ and HClO_4_ [23, 24]. The phosphorus concentrations in feed ingredients, diet samples and tibia ash were measured colorimetrically using the vanadate-molybdate method [25]. The phytate phosphorus (PP) concentrations of feed ingredients and diets were analyzed with the ferric precipitation method as described by Rutherfurd et al. [26] and then the dietary NPP was calculated with the formula to total phosphorus - PP. The concentrations of CP in feed ingredient and diet samples were determined using the methods of Association of Official Analytical Chemists (1990) [27].

### Determination of the tibia bone mineral concentration, bone mineral density, bone breaking strength, ash concentrations and the total phosphorus accumulation in tibia ash

The frozen tibia was thawed at room temperature for 2 h and then stripped of all soft tissues. The tibia bone mineral concentration (BMC) and bone mineral density (BMD) were then determined using Dual-Energy X-Ray Absorptiometry (DEXA, Tokyo, Japan) [12, 28]. The tibia bone breaking strength (BBS) of broilers on d 14, 28, and 42 was determined by a 3-point bending test (HDP/3 PB Texture Analyzers, UK) [12, 29]. To determine tibia ash concentration, the tibia was dried at 105 °C for 24 h, and then defatted with fresh diethyl ether for 48 h, and finally the fat-free, dried bone was ashed in a muffle furnace at 550 °C for 16 h [28]. The total phosphorus accumulation in tibia ash (TP_TA_) was calculated as described in our previous study [12].

### Determination of serum ca, phosphorus, ALP, FGF23, PTH, and 1,25(OH)_2_D_3_ concentrations and tibia ALP activity and BGP concentration

Serum was thawed and analyzed for the Ca concentration using a microplate reader with Ca assay kits (Nanjing Jiancheng Bioengineering Institute, Nanjing, China); serum phosphorus concentration was determined using the molybdenum blue method according to Goldenberg and Fernandez [30]; serum intact FGF23 concentration was assessed using an ELISA kit specially used for poultry according to the manufacturer’s protocol (Nanjing Jiancheng Bioengineering Institute, Nanjing, China); serum PTH level was measured using an ELISA kit according to the manufacturer’s protocol (Nanjing Jiancheng Bioengineering Institute, Nanjing, China); and serum 1,25(OH)_2_D_3_ level was measured by an ELISA kit by Beijing Sino-UK Institute of Biological Technology. The ALP activities in serum and tibia were measured using a microplate reader with ALP assay kits (Nanjing Jiancheng Bioengineering Institute, Nanjing, China). The tibia BGP concentration was determined by the method of ELISA with a commercial kit (Nanjing Jiancheng Bioengineering Institute, Nanjing, China).

### RNA isolation and RT-qPCR analysis

Total RNA from the tibia bone was isolated using TRIzol reagent (catalogue no. 15596018; Life Technologies) and reversely transcribed into complementary DNA (cDNA) according to the manufacturer’s instructions. The concentration of total RNA was estimated by measuring its optical density at 260 and 280 nm with a spectrophotometer (ND-100; NanoDrop Technologies), and the purity was determined in agarose gels stained with ethidium bromide. Then 500 ng of total RNA was reversely transcribed into cDNA using PrimeScript TM RT Master Mix (catalogue no. RR047A; TaKaRa Bio Inc.) according to the manufacturer’s instructions. cDNA was used as templates for real-time quantitative PCR machine following the manufacturer’s instructions. The PCR protocol was as follow: denaturation at 95 °C for 2 min followed by 40 cycles of 40 cycles of 94 °C for 15 s and 60 °C for 1 min. The primer sequences are listed in Supplementary Table S2. The mRNA level of the target gene was calculated using the 2^-△△Ct^ method, and the geometric mean of internal references, β-actin and glyceraldehydes 3-phosphate dehydrogenase (GAPDH) were used to normalize the expression of the targeted genes [31, 32].

### Western blot analysis

Tibia protein was isolated from liquid nitrogen snap frozen tibia by pulverizing the bone using a mortar and pestle and transferring the powder (40 mg) to 0.4 mL of ice-cold radio immunoprecipitation assay (RIPA) lysis buffer (catalogue no. P0013B, Beyotime Institute of Biotechnology) supplemented with 4 μL of protease inhibitor (catalogue no. B14001; BioTool). The lysates were sonicated for 10 s and 8 times in an ice-cold water bath and centrifuged to separate the protein extract, and the supernatant was collected for total protein determination using a bicinchoninic acid protein assay kit (catalogue no. 23225; Pierce). The extracted protein (30 μg) was subjected to electrophoresis on a 10% SDS-PAGE gel, and then electrotransferred onto the polyvinylidenefluoride membranes (catalogue no. IVPH00010; Merck-Millipore). After the transfer, membranes were blocked for 1 h at room temperature in a blocking buffer with 5% skimmed milk, and then incubated overnight at 4 °C with the following primary antibodies: FGF23 (catalogue no. A6124; Abclonal), PHEX (catalogue no. A7918; Abclonal), MEPE (catalogue no. ab108073; abcam), DMP1 (catalogue no. ab103203; abcam) and β-actin (catalogue no. HX1827; Huaxingbio). After several washes in tris-buffered saline with tween, membranes were incubated with the secondary antibody of goat anti-rabbit (catalogue no. HX2030; Huaxingbio) or goat anti-mouse (catalogue no. HX2032; Huaxingbio) for 1 h at room temperature. The signals were recorded with a chemiluminescence image scanner (Tanon, Shanghai, China) by enhanced chemiluminescence using a Super Signal West Pico Trail Kit (catalogue no. 34077; Pierce). Data were presented as the ratio of FGF23, PHEX, MEPE or DMP1 protein band intensity to β-actin protein band intensity. The β-actin protein was used to normalize the expression level of FGF23, PHEX, MEPE or DMP1.

### Statistical analyses

Data from the present study were analyzed by SAS statistical software (version 9.4, SAS Institute Inc., Cary, NC). The data were firstly tested for normality and homogeneity of variance using the Shapiro-Wilk and F-tests, respectively. Then the data were subjected to one-way ANOVA using the general liner model procedure, and the treatment comparisons for significant differences in all figures were tested by the least significant difference method. Orthogonal polynomials were applied for testing linear and quadratic effects of dependent variables to independent variables. Simple correlation analyses between the bone phosphorus retention or bone development parameters and related hormones or local bone-derived regulators were carried out based on replicate values from different NPP level treatments at each sampling time point using the Pearson procedure [12, 33]. The degree of correlation (strong correlation: ± 0.50 and ± 1; moderate correlation: ± 0.30 and ± 0.49; weak correlation: below ±0.29; and no correlation: 0) was determined as described by Chatterjee et al. [34]. The replicate cage was regarded as an experimental unit. The statistical significance was set at *P* ≤ 0.05.

## Results

### Growth performance and mortality

**Fig. 1:**
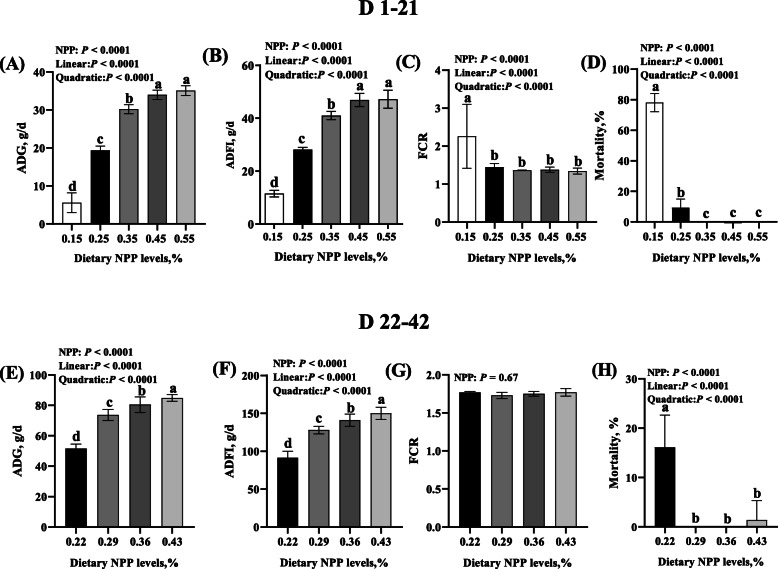
Effect of dietary NPP level on growth performance and mortality of broilers. **A** ADG from 1 to 21 days of age. **B** ADFI from 1 to 21 days of age. **C** FCR from 1 to 21 days of age. **D** Mortality from 1 to 21 days of age. **E** ADG from 22 to 42 days of age. **F** ADFI from 22 to 42 days of age. **G** FCR from 1 to 21 days of age. **H** Mortality from 22 to 42 d of age. All values are expressed as means±SD; Mortality, Percentage data for mortality of birds were transformed to arcsine for analysis; *n* = 8 for all of other groups except for *n* = 2 for the first group with 0.15% dietary NPP from d 1 to 21. Lacking the same letters (a, b, c, d) means differences at *P* < 0.0001. ADG, average daily gain; ADFI, average daily feed intake; feed conversion ratio (FCR); NPP, non-phytate phosphorus

Dietary NPP level affected (*P* < 0.0001) ADG, ADFI, and mortality of broilers from 1 to 21 and 22 to 42 days of age, as well as FCR of broilers from 1 to 21 days of age, but did not affect (*P* = 0.67) FCR of broilers from 22 to 42 days of age (Fig. 1). From 1 to 21 days of age, the ADG and ADFI increased while FCR decreased linearly and quadratically (*P* < 0.0001) as dietary NPP level increased (Fig. 1a-c). From 22 to 42 days of age, the ADG and ADFI increased linearly and quadratically (*P* < 0.0001) as dietary NPP level increased (Fig. 1e and f). The mortality of broilers from 1 to 21 or 22 to 42 days of age decreased linearly and quadratically (*P* < 0.0001) as dietary NPP level increased (Fig. 1d and h). Most of the broilers fed the diet with 0.15% dietary NPP died at 22 days of age, and thus we terminated this treatment from d 22 on, and continued the experiment with all of other four treatments.

### Tibia BMC, BMD, BBS, and ash concentration

**Fig. 2:**
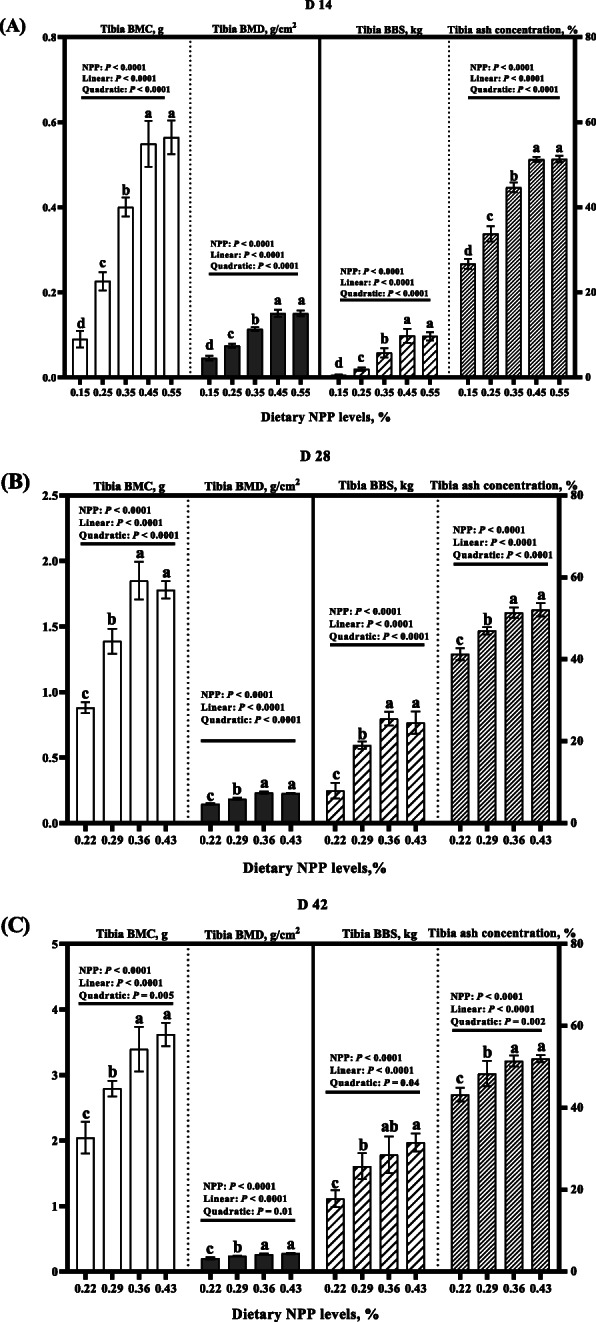
Effect of dietary NPP level on the traits of bone development of broilers. **A** Tibia BMC, BMD, BBS and ash concentrations at 14 d of age. **B** Tibia BMC, BMD, BBS and ash concentrations at 28 days of age. **C** Tibia BMC, BMD, BBS and ash concentrations at 42 days of age. All values are expressed as means ± SD; *n* = 7/8 per group. Lacking the same letters (a, b, c) means differences at *P* < 0.05. NPP, non-phytate phosphorus; BMC, bone mineral concentration; BMD, bone mineral density; BBS, bone breaking strength

Dietary NPP level affected (*P* < 0.0001) tibia BMC, BMD, BBS and ash concentrations of broilers at 14, 28 and 42 days of age (Fig. 2). All of the above indices at 14, 28 and 42 days of age increased linearly and quadratically (*P* < 0.05) as dietary NPP level increased (Fig. 2). At 14 days of age, all of the above indices increased gradually (*P* < 0.007) at 0.15–0.45% dietary NPP, and then reached a plateau (*P* > 0.37) at 0.45–0.55% dietary NPP (Fig. 2a). At 28 and 42 days of age, all of the above indices increased gradually (*P* < 0.004) at 0.22–0.36% dietary NPP, and then reached a plateau (*P* > 0.06) at 0.36–0.43% dietary NPP (Fig. 2b and c).

### Serum indices

**Fig. 3:**
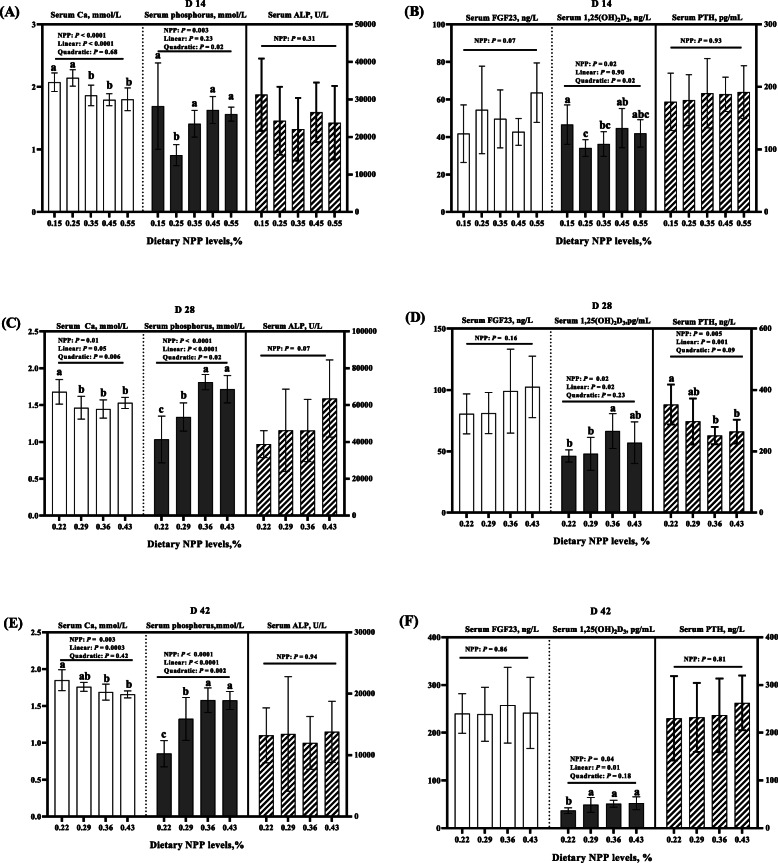
Effect of dietary NPP level on serum Ca and Phosphorus concentrations, ALP activity and related hormones of broilers. **A** The serum Ca, phosphorus concentrations and the activity of serum ALP at 14 days of age. **B** The serum FGF23, 1,25(OH)_2_D_3_ and PTH concentrations at 14 days of age. **C** The serum Ca, phosphorus concentrations and the activity of serum ALP at 28 days of age. **D** The serum FGF23, 1,25(OH)_2_D_3_ and PTH concentrations at 28 days of age. **E** The serum Ca, phosphorus concentrations and the activity of serum ALP at 42 days of age. **F** The serum FGF23, 1,25(OH)_2_D_3_ and PTH concentrations at 42 days of age. All values are expressed as means ± SD, *n* = 7/8 per group. Lacking the same letters (a, b, c) means differences at *P* ≤ 0.05. Ca, calcium; ALP, alkaline phosphatase; FGF23, Fibroblast growth factor 23; PTH, parathyroid hormone; 1,25(OH)_2_D_3_, 1,25-dihydroxyvitamin D3; NPP, non-phytate phosphorus

Dietary NPP level affected serum Ca, phosphorus and 1, 25(OH)_2_D_3_ concentrations at 14, 28 and 42 days of age (*P* < 0.05), and serum PTH concentrations at 28 days of age (*P* = 0.005), but did not affect serum ALP activity and FGF23 concentrations of broilers at 14, 28 and 42 days of age (*P* > 0.06), and serum PTH concentrations at 14 and 42 days of age (*P* > 0.80) (Fig. 3). At 14 and 42 days of age, serum Ca concentration decreased linearly (*P* < 0.0004), but at 28 days of age, it decreased linearly (*P* = 0.05) and quadratically (*P* = 0.006) as dietary NPP level increased (Fig. 3a, c and e). At 14 days of age, serum phosphorus and 1, 25(OH)_2_D_3_ concentration decreased quadratically (*P* = 0.02) (Fig. 3a and b), however, at 28 and 42 days of age, serum phosphorus increased linearly and quadratically (*P* < 0.03), and serum 1, 25(OH)_2_D_3_ increased linearly (*P* < 0.03) as dietary NPP level increased (Fig. 3c-f). At 28 days of age, serum PTH concentrations decreased linearly (*P* = 0.001) as dietary NPP level increased (Fig. 3d).

### Tibia ash ca and phosphorus concentrations, TP_TA_, tibia ALP activity and BGP concentration

**Fig. 4:**
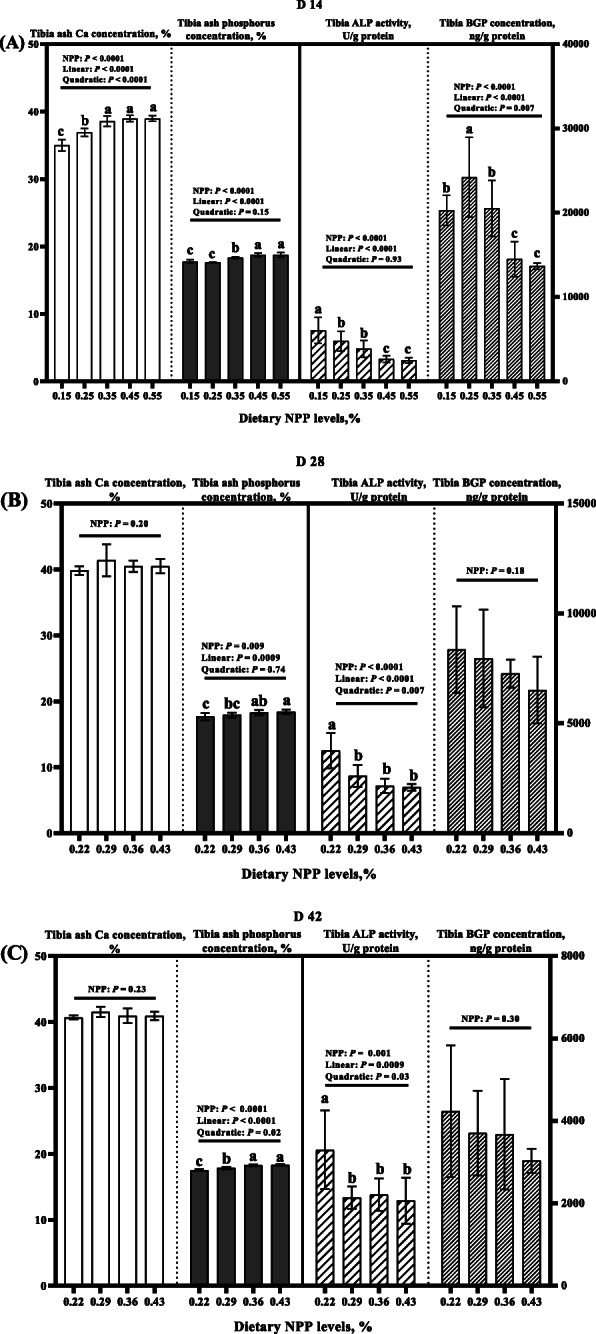
Effect of dietary NPP level on Ca, Phosphorus and BGP concentrations and ALP activity in the tibia of broilers. **A** The tibia ash Ca and phosphorus concentrations and tibia BGP concentration and ALP activity at 14 days of age. **B** The tibia ash Ca and phosphorus concentrations and tibia BGP concentration and ALP activity at 28 days of age. **C** The tibia ash Ca and phosphorus concentrations and tibia BGP concentration and ALP activity at 42 days of age. All values are expressed as means ± SD, *n* = 7/8 per group. Lacking the same letters (a, b, c) means differences at *P* ≤ 0.05. Ca, calcium; ALP, alkaline phosphatase, BGP, bone gal protein; NPP, non-phytate phosphorus

**Fig. 5:**
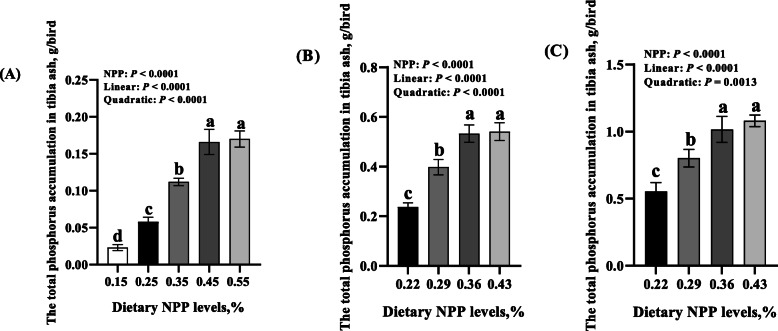
Effect of dietary NPP level on the total Phosphorus accumulation in the tibia ash of broilers. **A** the total phosphorus accumulation in tibia ash at 14 d of age. **B** the total phosphorus accumulation in tibia ash at 28 days of age. **C** the total phosphorus accumulation in tibia ash at 42 days of age. All values are expressed as means ± SD, *n* = 7/8 per group. Lacking the same letters (a, b, c, d) means differences at *P* ≤ 0.05

Dietary NPP level affected (*P* < 0.01) tibia ash phosphorus concentration, TP_TA_ and tibia ALP activity at 14, 28, and 42 days of age, as well as tibia ash Ca and tibia BGP concentrations at 14 days of age, but did not affect (*P* > 0.17) tibia ash Ca and tibia BGP concentrations on days 28 and 42 (Fig. 4 and Fig. 5). At 14 days of age, tibia ash Ca and TP_TA_ increased linearly and quadratically (*P* < 0.0001) and tibia BGP concentrations decreased linearly and quadratically (*P* < 0.008), while tibia ash phosphorus concentration increased linearly (*P* < 0.0001) and tibia ALP activity decreased linearly (*P* < 0.0001) as dietary NPP level increased (Fig. 4a and Fig. 5a). Tibia ash phosphorus concentration on d 28 increased linearly (*P* = 0.0009) and on d 42 it increased linearly and quadratically (*P* < 0.03), while tibia ALP activities on d 28 and 42 decreased linearly and quadratically (*P* < 0.04) as dietary NPP level increased (Fig. 4b and c). The TP_TA_ on d 28 and 42 increased linearly and quadratically (*P* < 0.002) as dietary NPP level increased (Fig. 5b and c).

### mRNA and protein expressions of local bone-derived regulators

**Fig. 6:**
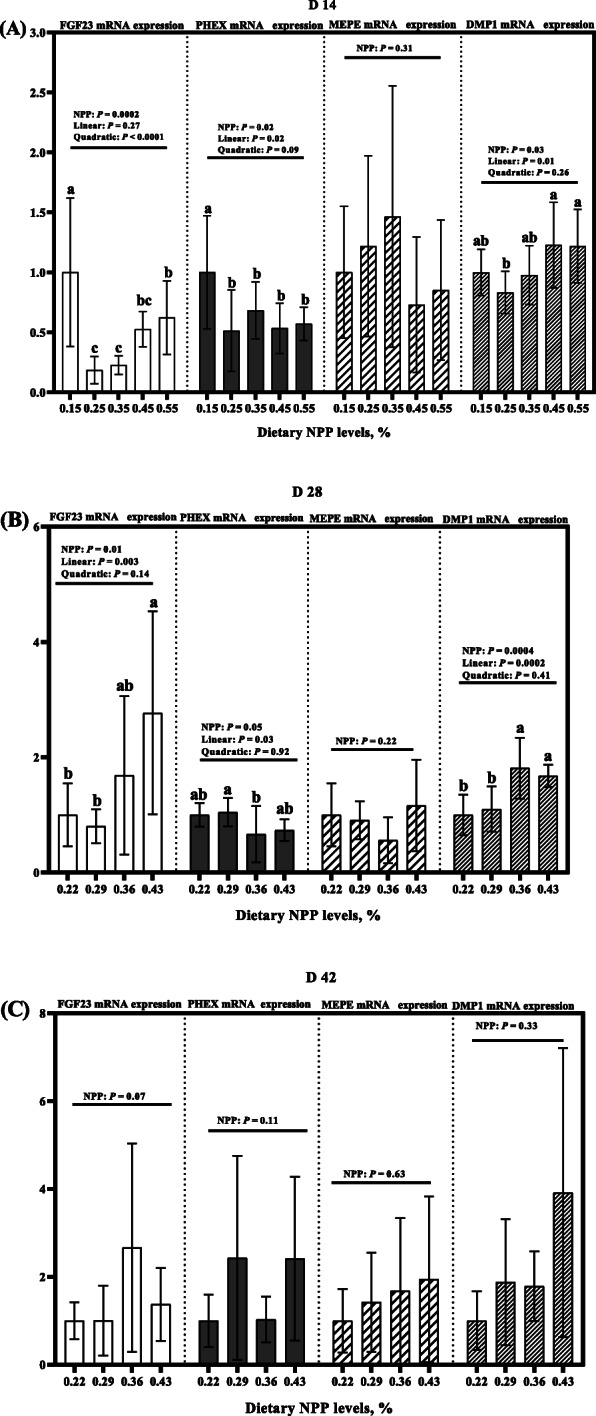
Effect of dietary NPP on mRNA expressions of local bone-derived regulators in the tibia of broilers. **A** FGF23, PHEX, MEPE, and DMP1 mRNA expressions at 14 d of age. **B** FGF23, PHEX, MEPE, and DMP1 mRNA expressions at 28 days of age. **C** FGF23, PHEX, MEPE, and DMP1 mRNA expressions at 42 days of age. All values are expressed as means ± SD, *n* = 7/8 per group. Lacking the same letters (a, b, c) means differences at *P* ≤ 0.05. FGF23, Fibroblast growth factor 23; PHEX, phosphate-regulating gene homologous to endopeptidase on X chromosome; MEPE, matrix extracellular phosphoglycoprotein; DMP1, Dentin matrix protein 1. The mRNA level was calculated as the ratio of target-gene mRNA to the geometric mean of *β-actin* and GAPDH mRNA, and RQ = 2^−ΔΔCT^ (CT = threshold cycle)

Dietary NPP level affected (*P* ≤ 0.05) *FGF23, PHEX* and *DMP1* mRNA expressions in the tibia at 14 and 28 d of age, but did not affect (*P* > 0.06) tibia *MEPE* mRNA expression at 14, 28 and 42 days of age and *FGF23, PHEX*, *MEPE* and *DMP1* mRNA expressions at 42 days of age (Fig. 6). At 14 and 28 days of age, tibia *FGF23* mRNA expression increased linearly or quadratically (*P* < 0.004), and tibia *PHEX* mRNA expression decreased linearly (*P* < 0.04), while tibia *DMP1* mRNA expression increased linearly (*P* < 0.02) as dietary NPP level increased (Fig. 6a and b).

**Fig. 7:**
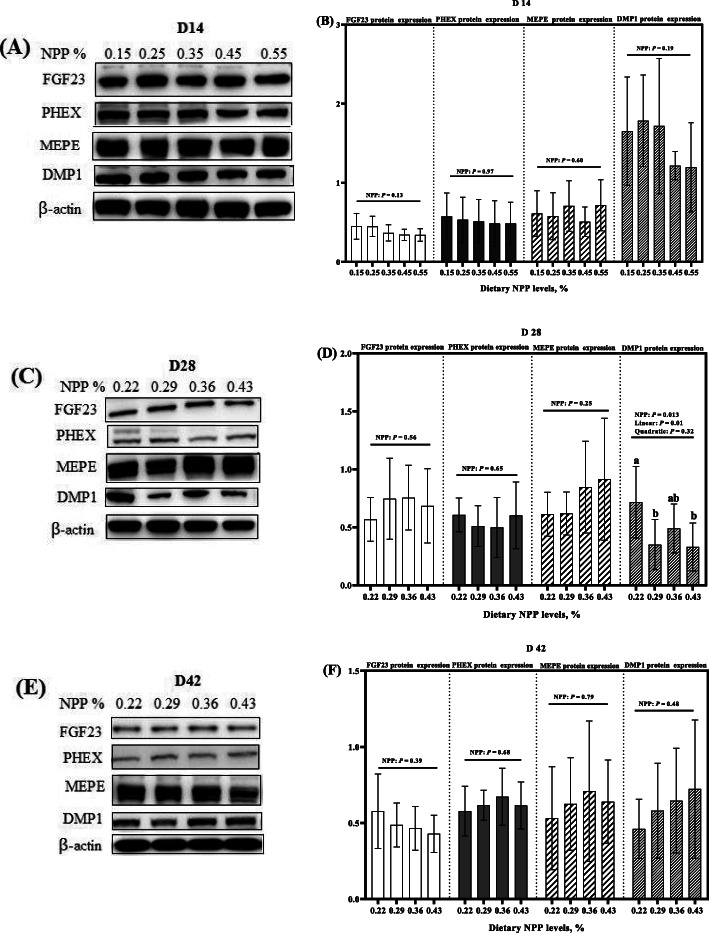
Effect of dietary NPP on protein expressions of local bone-derived regulators in the tibia of broilers. **A** Representative immunoblots of the indicated protein were shown at 14 days of age. **B** The data on protein expressions of FGF23, PHEX, MEPE, and DMP1 at 14 days of age. **C** Representative immunoblots of the indicated protein were shown at 28 days of age. **D** The data on protein expressions of FGF23, PHEX, MEPE, and DMP1 at 28 days of age. **E** Representative immunoblots of the indicated protein were shown at 42 days of age. **F** The data on protein expressions of FGF23, PHEX, MEPE, and DMP1 at 42 days of age. All values are expressed as means ± SD, *n* = 7/8 per group. Lacking the same letters (a, b) means differences at *P* < 0.05. FGF23, Fibroblast growth factor 23; PHEX, phosphate-regulating gene homologous to endopeptidase on X chromosome; MEPE, matrix extracellular phosphoglycoprotein; DMP1, Dentin matrix protein 1

Dietary NPP level affected (*P* < 0.02) tibia DMP1 protein expression at 28 days of age, but did not affect (*P* > 0.12) tibia FGF23, PHEX, MEPE, and DMP1 protein expressions at 14 and 42 days of age, as well as tibia FGF23, PHEX and MEPE protein expressions of broilers at 28 days of age (Fig. 7). At 28 days of age, tibia DMP1 protein expression decreased linearly (*P* = 0.01) as dietary NPP level increased (Fig. 7c and d).

### Correlations between bone phosphorus retention or bone development and serum related hormones

At 14 days of age, all of bone phosphorus retention or bone development parameters were not correlated (*P* > 0.22) with either 1, 25(OH)_2_D_3_ or PTH in serum (Table 1). At 28 days of age, TP_TA_ (*r* = 0.389, *P* = 0.028), tibia BMC (*r* = 0.425, *P* = 0.017), BMD (*r* = 0.461, *P* = 0.029), BBS (*r* = 0.393, *P* = 0.029), and ash concentration (*r* = 0.419, *P* = 0.019) were positively correlated with serum 1, 25(OH)_2_D_3_, but tibia ash phosphorus concentration was not correlated (*P* > 0.05) with serum 1,25(OH)_2_D_3_. In addition, tibia ash phosphorus concentration (*r* = − 0.538, *P* = 0.001), TP_TA_ (*r* = − 0.597, *P* < 0.001), BMC (*r* = − 0.590, *P* < 0.001), BMD (*r* = − 0.608, *P* < 0.001), BBS (*r* = − 0.609, *P* < 0.001), and ash concentration (r = − 0.587, *P* = 0.001) were negatively correlated with serum PTH. At 42 days of age, tibia ash phosphorus concentration (*r* = 0.486, *P* = 0.006), TP_TA_ (*r* = 0.450, *P* = 0.013), BMC (*r* = 0.422, *P* = 0.018), BMD (*r* = 0.430, *P* = 0.016), and ash concentration (*r* = 0.476, *P* = 0.019) were positively correlated with serum 1,25(OH)_2_D_3_, but tibia BBS was not correlated (*P* = 0.10) with serum 1, 25(OH)_2_D_3_. However, all of the above bone phosphorus retention or bone development parameters were not correlated (*P* > 0.10) with serum PTH on d 42.

**Table 1:** The correlation coefficients between bone phosphorus retention or bone development and serum related hormones in broilers at 14, 28 and 42 days of age ^a^

Traits	Serum 1, 25(OH)_**2**_D_**3**_	Serum PTH
D 14		
Tibia ash phosphorus concentration	0.198	0.073
TP_TA_	0.040	0.129
Tibia BMC	0.011	0.129
Tibia BMD	0.031	0.135
Tibia BBS	0.056	0.115
Tibia ash concentration	0.341	−0.300
D 28		
Tibia ash phosphorus concentration	0.013	−0.538**
TP_TA_	0.389*	−0.597**
Tibia BMC	0.425*	−0.590**
Tibia BMD	0.461**	−0.608**
Tibia BBS	0.393*	−0.609**
Tibia ash concentration	0.419*	−0.587**
D 42		
Tibia ash phosphorus concentration	0.486**	0.224
TP_TA_	0.450*	0.169
Tibia BMC	0.422*	0.220
Tibia BMD	0.430*	0.176
Tibia BBS	0.299	0.287
Tibia ash concentration	0.476*	0.089

^a^ Data represent the correlation coefficients between bone P retention or bone development and serum related hormones in broilers at 14 (*n* = 39/40), 28 and 42 (*n* = 31/32) d of age. * *P* < 0.05, ** *P* < 0.01. *TP*_*TA*_ The total phosphorus accumulation in tibia ash, *BMC* Bone mineral concentration, *BMD* Bone mineral density, *BBS* Bone breaking strength, *1,25(OH)*_*2*_*D*_*3*_ 1,25-dihydroxyvitamin D_3_, *PTH* Parathyroid hormone

Correlations between all of the above bone phosphorus retention or bone development parameters and serum FGF23 on d 14, 28 and 42 were not analyzed because serum FGF23 concentrations at these time points were not affected by dietary NPP level as shown above.

### Correlations between bone phosphorus retention or bone development and local bone-derived regulators

At 14 days of age, all of bone phosphorus retention or bone development parameters were not correlated (*P* > 0.10) with tibia *FGF23* mRNA expression (Table 2). On d 28, the tibia BMD (*r* = 0.384, *P* = 0.040) was positively correlated with the tibia *FGF23* mRNA expression, but all of the other bone phosphorus retention or bone development parameters were not correlated (*P* > 0.07) with tibia *FGF23* mRNA expression. On d 42, the tibia ash concentration (*r* = 0.389, *P* = 0.034) was positively correlated with the tibia *FGF23* mRNA expression, but all of the other bone phosphorus retention or bone development parameters were not correlated (*P* > 0.10) with the tibia *FGF23* mRNA expression. At 14 days of age, TP_TA_ (*r* = − 0.397, *P* = 0.013), tibia BMC (*r* = − 0.362, *P* = 0.024), and ash concentration (*r* = − 0.386, *P* = 0.015) were negatively correlated with the tibia *PHEX* mRNA expression, but tibia ash phosphorus, BMD and BBS were not correlated (*P* > 0.06) with the tibia *PHEX* mRNA expression. On d 28, the tibia BMD (*r* = − 0.395, *P* = 0.028) was negatively correlated with the tibia *PHEX* mRNA expression, but all of the other bone phosphorus retention or bone development parameters were not correlated (*P* > 0.05) with the tibia *PHEX* mRNA expression. On d 42, all of the bone phosphorus retention or bone development parameters were not correlated (*P* > 0.22) with the *PHEX* mRNA expression. At 14, 28 and 42 days of age, tibia ash phosphorus concentrations (*r* = 0.380 to 0.417, *P* < 0.03), TP_TA_ (*r* = 0.394 to 0.667, *P* < 0.03), BMC (*r* = 0.409 to 0.689, *P* < 0.02), BMD (r = 0.419 to 0.441, *P* < 0.02), BBS (*r* = 0.432 to 0.617, *P* < 0.02), and ash (*r* = 0.397 to 0.688, *P* < 0.04) were positively correlated with the tibia *DMP1* mRNA expression; the tibia ash phosphorus concentrations (*r* = − 0.697 to − 0.513, *P* < 0.005), TP_TA_ (*r* = − 0.828 to − 0.632, *P* < 0.001), BMC (*r* = − 0.803 to − 0.629, *P* < 0.001), BMD (*r* = − 0.806 to − 0.621, *P* < 0.001), BBS (*r* = − 0.807 to − 0.549, *P* < 0.003), and ash (*r* = − 0.810 to − 0.624, *P* < 0.001) were negatively correlated with the tibia ALP*.* At 14 days of age, tibia ash phosphorus concentrations (*r* = − 0.604, *P* < 0.001), TP_TA_ (*r* = − 0.660, *P* < 0.001), BMC (*r* = − 0.634, *P* < 0.001), BMD (*r* = − 0.651, *P* < 0.001), BBS (*r* = − 0.686, *P* < 0.001), and ash (*r* = − 0.643, *P* < 0.001) were negatively correlated with the tibia BGP. On d 28, TP_TA_ (*r* = − 0.425, *P* = 0.017), tibia BMC (*r* = − 0.401, *P* = 0.028), BMD (*r* = − 0.416, *P* = 0.020), and BBS (*r* = − 0.409, *P* = 0.025) were negatively correlated with the tibia BGP, but tibia ash phosphorus and ash were not correlated (*P* > 0.05) with the tibia BGP. On d 42, tibia BBS (*r* = − 0.362, *P* = 0.049) was negatively correlated with the tibia BGP, but all of the other bone phosphorus retention or bone development parameters were not correlated (*P* > 0.06) with the tibia BGP.

**Table 2:** The correlation coefficients bone phosphorus retention or bone development and local bone-derived regulators in broilers at 14, 28 and 42 days of age ^a^

Traits	*FGF23* mRNA expression	*PHEX* mRNA expression	*DMP1* mRNA expression	Tibia ALP	Tibia BGP
D 14					
Tibia ash phosphorus concentration	0.052	−0.299	0.380*	−0.697**	− 0.604**
TP_TA_	−0.198	− 0.397*	0.394*	− 0.828**	−0.660**
Tibia BMC	−0.222	−0.362*	0.409*	−0.803**	− 0.634**
Tibia BMD	−0.224	− 0.377	0.419**	−0.806**	− 0.651**
Tibia BBS	−0.143	− 0.303	0.432**	−0.774**	− 0.686**
Tibia ash concentration	−0.229	− 0.386*	0.401*	− 0.810**	−0.643**
D 28					
Tibia ash phosphorus concentration	0.068	0.087	0.471**	−0.513**	−0.083
TP_TA_	0.311	−0.333	0.667**	−0.763**	− 0.425*
Tibia BMC	0.295	−0.312	0.689**	−0.741**	− 0.401*
Tibia BMD	0.384*	−0.395*	0.617**	− 0.764**	−0.416*
Tibia BBS	0.339	−0.353	0.617**	−0.807**	− 0.409*
Tibia ash concentration	0.291	−0.283	0.688**	− 0.800**	−0.361
D 42					
Tibia ash phosphorus concentration	0.312	0.158	0.437*	−0.515**	−0.310
TP_TA_	0.314	0.138	0.420*	−0.632**	−0.325
Tibia BMC	0.291	0.169	0.462*	−0.629**	−0.343
Tibia BMD	0.284	0.205	0.441*	−0.621**	− 0.317
Tibia BBS	0.280	0.218	0.442*	−0.549**	−0.362*
Tibia ash concentration	0.389*	0.268	0.397*	−0.624**	−0.303

^a^ Data represent the correlation coefficients between bone phosphorus retention or bone development and local bone-derived regulators in broilers at 14 (*n* = 39/40), 28 and 42 (*n* = 31/32) days of age. *P < 0.05, **P < 0.01. *TP*_*TA*_ the total phosphorus accumulation in tibia ash, *BMC* bone mineral concentration, *BMD* bone mineral density, *BBS* bone breaking strength, *FGF23* Fibroblast growth factor 23, *PHEX* phosphate-regulating gene homologous to endopeptidase on X chromosome, *DMP1* Dentin matrix protein 1, *ALP* alkaline phosphatase, *BGP* bone gal protein

Correlations between all of the bone phosphorus retention or bone development parameters and protein expressions of the above local bone-derived regulators or MEPE mRNA and protein expressions on d 14, 28 and 42 were not analyzed because protein expressions of the above local bone-derived regulators or MEPE mRNA and protein expressions at these time points were not affected by dietary NPP level except for the tibia DMP1 protein expression on d 28 as shown above.

## Discussion

Our hypothesis that bone phosphorus retention and bone development might be regulated not only by the related hormones, but also by the local bone-derived factors in broiler chickens has been supported by the results of the present study. The results from the present study suggested that bone phosphorus retention and bone development might partly be regulated by serum PTH and 1,25(OH)_2_D_3_, tibia FGF23, PHEX, DMP1, ALP and BGP in broiler chickens during the whole growth period. Therefore, these above findings could help us to better understand the mechanisms of bone phosphorus retention and bone development, and provided novel and scientific experimental bases for improving bone phosphorus utilization in broiler chickens.

The results from the present study also indicate that phosphorus plays an important role in promoting the growth, bone development and mineralization in the fast-growing broiler chickens. In the current study, the ADG and ADFI, tibia BMD, BMC, BBS and ash concentrations of broiler chickens were significantly improved by the increase of dietary NPP level in the whole growth period, which is similar to the results reported from our laboratory [21, 35]. Therefore, broiler chickens are very susceptible to dietary phosphorus deficiency, and they could be used as an ideal animal model for studying the bone phosphorus metabolism and bone development and health. However, there were no huge mortality rates for the broilers fed the diets with similar deficient levels of dietary NPP in our previous two studies [35, 36]. The huge mortality rate of broilers fed the diet with 0.15% dietary NPP in the present study might be associated with a very high susceptibility of these birds to dietary phosphorus deficiency. Exact reasons remain to be further studied.

Additionally, many studies have indicated that the ratio of Ca:NPP is more important for growth and bone development of broilers than the absolute concentration of either Ca or NPP [37, 38]. It is reported that a decrease in the dietary levels of Ca and NPP at a constant Ca:NPP ratio (about 2.0:1) improved the FCR, without affecting the growth rate of broilers, but impaired the bone development [39]. Therefore, it needs to be further studied how the results would change if the Ca:NPP ratio had been maintained at a constant ratio (e.g. 1.94:1).

The phosphorus homeostasis is critical for many cellular processes and bone mineralization, which is tightly regulated by several related hormones [40, 41]. The classical calcitropic hormones, such as PTH and 1,25(OH)_2_D_3_, as well as FGF23, regulates systemic phosphorus levels by creating bone-kidney-parathyroid feedback loops [42]. The PTH is secreted by the parathyroid glands and is the most important regulator of phosphorus levels in the blood and bones [43]. A previous study has shown that a high phosphorus diet increased PTH secretion and then caused an ongoing process in bone reabsorption in rats [44], and PTH can induce the synthesis of 1,25(OH)_2_D_3_ in the kidney, and then in turn, 1,25(OH)_2_D_3_ can inhibit PTH secretion by the parathyroid glands [45]. In vehicle-treated hypophosphatemic mice, the serum 1,25(OH)_2_D_3_ concentration was inappropriately low for the degree of hypophosphatemia, compared with values in wild type mice [46], and patients with hypophosphatemia show inappropriately low serum 1,25(OH)_2_D_3_ level [47]. In our study, serum 1,25(OH)_2_D_3_ concentrations on d 28 and 42 increased as dietary NPP level increased and had moderate correlations with most of bone phosphorus retention or bone development parameters. Serum PTH concentration on d 28 decreased as dietary NPP level increased and had strong correlations with all of bone phosphorus retention or bone development parameters. The above results from the present study suggested that the two hormones 1,25(OH)_2_D_3_ and PTH might regulate bone phosphorus retention and bone development of broilers. However, in the current study, serum FGF23 was not affected by dietary NPP level, which might be due to the concomitant changes in these parameters, such as serum phosphorus and Ca that clearly affected FGF23 levels. It is thus speculated that serum PTH, 1,25(OH)_2_D_3_ and FGF23 are controlled within a narrow physiological range by feedback mechanisms and their respective receptors between bone and other organs, and exact mechanisms and their relationships need to be further studied.

The local bone-derived regulators are not only markers of osteocyte maturation, but also functionally involved in phosphorus regulation and bone mineralization [48]. The DMP1 and PHEX are known to be involved in inhibition of FGF23 production [49]. Wang et al. (2018) reported that dietary high phosphorus level up-regulated *FGF23* mRNA expression in tibia compared with dietary low phosphorus level in broilers [17]. A study showed that an increased level of inorganic phosphorus in media up-regulated the DMP1 protein expression in primary osteocytes isolated from mouse bones [8]. A study also reported that *MEPE* mRNA expression was up-regulated in bone and osteoblasts derived from hypophosphatemia mice [50]. The PHEX is the gene whose mutation is the cause of X-linked hypophosphatemia in humans and mice, and their possible sensitivity to stimulation by low phosphorus diets [51]. In the present study, tibia *FGF23* and *DMP1* mRNA expressions increased and *PHEX* mRNA expression decreased on d 14 or 28 as dietary NPP level increased, while tibia *MEPE* mRNA expression was not affected, but tibia DMP1 protein expression on d 28 decreased as dietary NPP level increased, and protein expressions of DMP1 on d 14 and 42 and the other local bone-derived regulators in tibia of broilers were not affected by dietary NPP. It is noted that protein expressions of tibia FGF23, PHEX and DMP1 were inconsistent with their mRNA expressions on d 14 and 28, which might be due to the effects of some factors, such as translation rate, post-translational modification, greater biological and experimental variations [35], and so on. Exact reasons need to be further studied. In addition, some studies showed that the ALP and BGP are closely related to the rate of bone mineralization in broilers [13, 52]. The ALP functions mainly by degrading inorganic pyrophosphate and providing free inorganic phosphorus, and the tissue nonspecific ALP has a positive influence on mineralization primarily by controlling the size of the inhibitory pool of pyrophosphate through its inorganic pyrophosphatase activity [53, 54]. The ALP can be secreted by osteoblasts and regarded as an important component of osteoblastic activity and osteogenesis [55]. Hence, the increase of ALP activity indicated that the bone tissue underwent more intense osteogenesis [56]. However, the increased inorganic phosphorus resulting from the increased ALP activity can be also used for other metabolic functions, such as energy metabolism, cellular signaling, membrane integrity, enzyme activity, and so on [35]. The BGP is a specific product of the osteoblast involved in bone formation and inhibits the hydroxyapatite formation, and thus relatively low concentrations of BGP are present in bone matrix at the onset of mineralization [57, 58]. In our current study, tibia ALP activities on d 14, 28 and 42 decreased as dietary NPP levels increased, which is similar to previous reports from our laboratory [21, 35], suggesting that birds might obtain phosphorus for important metabolic functions (energy metabolism, cellular signaling, membrane integrity, enzyme activity, etc.) through increasing the bone ALP activity or bone resorption when dietary phosphorus is below the requirement [35]. The tibia BGP concentrations on d 14 decreased as dietary NPP levels increased, but dietary NPP level did not affect the tibia BGP concentrations of broilers on d 28 and 42, suggesting that the BGP might play a more important role in the starter broilers than in the grower broilers.

Moreover, in the present study, all of bone phosphorus retention or bone development parameters on d 14, 28 and 42 had moderate or strong correlations with tibia *DMP1* mRNA expression, and TP_TA_, tibia BMC and ash concentration on d 14 and tibia BMD on d 28 had moderate correlations with *PHEX* mRNA expression. Tibia BMD on d 28 and ash concentration on d 42 had moderate correlations with tibia *FGF23* mRNA expression. However, all of bone phosphorus retention or bone development parameters had moderate or strong correlations with tibia ALP on d 14, 28 and 42, and tibia BGP on d 14. The above results suggested that bone phosphorus retention and bone development might also be regulated by these local bone-derived regulators (DMP1, PHEX, FGF23, ALP and BGP) of broiler chickens in the whole growth period. To our knowledge, this has been not reported before. However, further studies need be carried out to verify the roles of these local bone-derived regulators in regulating bone phosphorus retention and bone development of broiler chickens.

## Conclusions

The results from the present study indicated that adequate dietary phosphorus is essential for enhancing the growth, phosphorus homeostasis in serum or tibia and bone mineralization of fast-growing broiler chickens; and bone phosphorus retention and bone development parameters had moderate to strong correlations with serum PTH and 1,25(OH)_2_D_3_, tibia DMP1, PHEX, FGF23, ALP and BGP in broilers during the whole growth period, and thus they might be partly regulated by these related hormones and local bone-derived regulators. Further studies warrant to be done to verify the roles of these hormones and local bone-derived regulators in regulating bone phosphorus retention and bone development of broiler chickens, and how the results would change if the Ca:NPP ratio had been maintained at a constant ratio (e.g. 1.94:1).

## Supplementary Information

**Additional file 1: Supplementary Table S1.** Composition and nutrient levels of the experimental diets for broilers ^a^ (as-fed basis). **Supplementary Table S2.** Primer sequences for RT-qPCR amplification ^a^.

## Data Availability

The data are shown in the main manuscript and supplemental materials.

## Abbreviations

ADFI: Average daily feed intake
ADG: Average daily gain
ALP: Alkaline phosphatase
BBS: Bone breaking strength
BGP: Bone gal protein
BMC: Tibia bone mineral concentration
BMD: Bone mineral density
Ca: Calcium
CP: Crude protein
Ca: NPP (Calcium: Non-phytate phosphorus)
DMP1: Dentin matrix protein 1
FCR: Feed conversion ratio
FGF23: Fibroblast growth factor 23
GAPDH: Glyceraldehydes 3-phosphate dehydrogenase
1,25(OH)_2_D_3_: 1,25-dihydroxyvitamin D_3_
PHEX: Phosphate-regulating gene with homologies to endopeptidases on the X chromosome
PP: Phytate phosphorus
PTH: Parathyroid hormone
MEPE: Matrix extracellular phosphoglycoprotein
TP_TA_: The total phosphorus accumulation in tibia ash
