# Supplementary material for: Regulation of bone phosphorus retention and bone development possibly by related hormones and local bone-derived regulators in broiler chicks
Source: J Anim Sci Biotechnol. 2021 Aug 12;12:88. doi: 10.1186/s40104-021-00610-1 (PMC8359065; doi:10.1186/s40104-021-00610-1)
Supplement: Supplementary file 1 — Additional file 1: Supplementary Table S1. Composition and nutrient levels of the experimental diets for broilers a (as-fed basis). Supplementary Table S2. Primer sequences for RT-qPCR amplification a. [file 40104_2021_610_MOESM1_ESM.doc]

| **Item** | **Starter (D 1 to 21)** | | | | |  | **Grower (D 21 to 42)** | | | | |
| --- | --- | --- | --- | --- | --- | --- | --- | --- | --- | --- | --- |
|  | **Dietary NPP levels, %** | | | | |  | **Dietary NPP levels, %** | | | | |
|  | **0.15** | **0.25** | **0.35** | **0.45** | **0.55** |  | **0.15** | **0.22** | **0.29** | **0.36** | **0.43** |
| Ingredients, % |  |  |  |  |  |  |  |  |  |  |  |
| Ground yellow corn | 50.3 | 50.3 | 50.3 | 50.3 | 50.3 |  | 58.3 | 58.3 | 58.3 | 58.3 | 58.3 |
| Soybean meal | 40.7 | 40.7 | 40.7 | 40.7 | 40.7 |  | 33.4 | 33.4 | 33.4 | 33.4 | 33.4 |
| Soybean oil | 4.88 | 4.88 | 4.88 | 4.88 | 4.88 |  | 4.87 | 4.87 | 4.87 | 4.87 | 4.87 |
| Salt ^b^ | 0.30 | 0.30 | 0.30 | 0.30 | 0.30 |  | 0.30 | 0.30 | 0.30 | 0.30 | 0.30 |
| _DL_-methionine | 0.24 | 0.24 | 0.24 | 0.24 | 0.24 |  | 0.16 | 0.16 | 0.16 | 0.16 | 0.16 |
| Micronutrients ^b,c^ | 0.32 | 0.32 | 0.32 | 0.32 | 0.32 |  | 0.21 | 0.21 | 0.21 | 0.21 | 0.21 |
| CaHPO_4_**^.^**H_2_O ^b^ | 0.00 | 0.58 | 1.16 | 1.74 | 2.32 |  | 0.00 | 0.41 | 0.81 | 1.22 | 1.62 |
| Limestone ^b^ | 2.22 | 1.91 | 1.60 | 1.29 | 0.98 |  | 2.03 | 1.82 | 1.60 | 1.38 | 1.17 |
| Sand | 1.08 | 0.81 | 0.54 | 0.27 | 0.00 |  | 0.76 | 0.57 | 0.38 | 0.19 | 0.00 |
| Nutrient composition, % |  |  |  |  |  |  |  |  |  |  |  |
| Metabolizable energy, MJ/kg | 12.5 | 12.5 | 12.5 | 12.5 | 12.5 |  | 12.9 | 12.9 | 12.9 | 12.9 | 12.9 |
| Crude protein ^d^ | 22.2 | 22.3 | 22.3 | 22.4 | 22.3 |  | 19.8 | 19.9 | 19.7 | 19.8 | 19.8 |
| Lysine | 1.10 | 1.10 | 1.10 | 1.10 | 1.10 |  | 1.02 | 1.02 | 1.02 | 1.02 | 1.02 |
| Methionine | 0.55 | 0.55 | 0.55 | 0.55 | 0.55 |  | 0.43 | 0.43 | 0.43 | 0.43 | 0.43 |
| Methionine +cysteine | 0.90 | 0.90 | 0.90 | 0.90 | 0.90 |  | 0.73 | 0.73 | 0.73 | 0.73 | 0.73 |
| Calcium ^d^ | 1.02 | 1.04 | 1.00 | 1.03 | 1.03 |  | 0.84 | 0.87 | 0.88 | 0.88 | 0.89 |
| Total phosphorus ^d^ | 0.41 | 0.51 | 0.61 | 0.71 | 0.81 |  | 0.39 | 0.46 | 0.54 | 0.61 | 0.68 |
| Non-phytate phosphorus ^d^ | 0.14 | 0.24 | 0.34 | 0.43 | 0.53 |  | 0.14 | 0.20 | 0.27 | 0.31 | 0.40 |

**Supplementary Table S1** Composition and nutrient levels of the experimental diets for broilers ^a^ (as-fed basis)

^a^ Each diet was formulated by changing the quantities of fine sand, limestone, and CaHPO_4_^.^H_2_O. Washed fine sand contained no detectable phosphorus and Ca.

^b^ Feed grade.

^c^ For starter diets, provided per kilogram of diet: 15,000 IU vitamin A (all trans-retinol acetate); 4,500 IU cholecalciferol; 24 IU vitamin E (all-rac-α-tocopherol acetate); 3 mg vitamin K (menadione sodium bisulfate); 3 mg thiamin (thiamin mononitrate); 9.6 mg riboflavin; 3 mg vitamin B_6_; 0.018 mg vitamin B_12_; 15 mg calcium pantothenate; 39 mg niacin; 1.5 mg folic acid; 0.15 mg biotin; 700 mg choline (choline chloride); 60 mg Zn (ZnSO_4_·7H_2_O); 8 mg Cu (CuSO_4_ 5H_2_O); 110 mg Mn (MnSO_4_·H_2_O); 40 mg (FeSO_4_·7H_2_O); 0.35 mg I (KI); 0.15 mg Se (Na_2_SeO_3_); 50 mg chlorotetracy cline. For grower diets, provided per kilogram of diet: 10,000 IU vitamin A (all trans-retinol acetate); 3,000 IU cholecalciferol; 16 IU vitamin E (all-rac-α-tocopherol acetate); 2 mg vitamin K (menadione sodium bisulfate); 2 mg thiamin (thiamin mononitrate); 6.4 mg riboflavin; 2 mg vitamin B_6_; 0.012 mg vitamin B_12_; 10 mg calcium pantothenate; 26 mg niacin; 1 mg folic acid; 0.1 mg biotin; 500 mg choline (choline chloride); 40 mg Zn (ZnSO_4_·7H_2_O); 8 mg Cu (CuSO_4_ 5H_2_O); 80 mg Mn (MnSO_4_·H_2_O); 30 mg (FeSO_4_·7H_2_O); 0.35 mg I (KI), 0.15 mg Se (Na_2_SeO_3_).

^d^ Analyzed values. Each value based on triplicate determinations. The others were calculated values.

**Supplementary Table S2.** Primer sequences for RT-qPCR amplification ^a^

| **Gene** | **GenBank ID** | **PCR products, bp** | **Primer sequences** |
| --- | --- | --- | --- |
| *β-actin* | NM_205518.1 | 95 | F: 5’-ACCTGAGCGCAAGTACTCTGTCT-3’ |
|  |  |  | R: 5’-CATCGTACTCCTGCTTGCTGAT-3’ |
| GAPDH | NM_204305.1 | 128 | F: 5’-CTTTGGCATTGTGGAGGGTC-3’ |
|  |  |  | R: 5’-ACGCTGGGATGATGTTCTGG-3’ |
| FGF23 | XM425663.2 | 190 | F: 5’-ATGCTGCTTGTGCTCTGTATC-3’ |
|  |  |  | R: 5’-ACTGTAAATGGTTTGGTGAGG-3’ |
| PHEX | NM_001199277.2 | 194 | F: 5’-TTTACGAGCCCAACTACTGT-3’ |
|  |  |  | R: 5’-TTGTCATATTTCCGACCAT-3’ |
| MEPE | NM_204569.1 | 193 | F: 5’-AAAGGGTTGCCATAGGAAAGTC-3’ |
|  |  |  | R: 5’-TCAGCGTGCCTCACCTGCTC-3’ |
| DMP1 | NM_206993.1 | 226 | F: 5’-ACGCTTCTACACCTCTGCTG-3’ |
|  |  |  | R: 5’-CTACGTCCGCATCACCAGT-3’ |

^a^ GAPDH, glyceraldehyde-3-phosphate dehydrogenase; FGF23, fibroblast growth factor 23; PHEX, phosphate-regulating gene homologous to endopeptidase on X chromosome; MEPE, matrix extracellular phosphoglycoprotein; DMP1, dentin matrix protein 1. F = forward; R = reverse.
